# Neuroimaging and cognitive correlates of retinal Optical Coherence Tomography (OCT) measures at late middle age in a twin sample

**DOI:** 10.1038/s41598-022-13662-8

**Published:** 2022-06-10

**Authors:** Chris Moran, Zheng Yang Xu, Hemal Mehta, Mark Gillies, Chris Karayiannis, Richard Beare, Christine Chen, Velandai Srikanth

**Affiliations:** 1 National Centre for Healthy Ageing, Melbourne, Australia; 2 Department of Geriatric Medicine, Peninsula Health and Central Clinical School, Monash University, Melbourne, Australia; 3 Department of Aged Care, Alfred Health, Melbourne, Australia; 4 Royal Free London NHS Foundation Trust, London, UK; 5 UCL Medical School, London, UK; 6 Macular Research Group, University of Sydney, Sydney, Australia; 7 Department of Ophthalmology, Monash Health, Melbourne, Australia

**Keywords:** Cognitive ageing, Cognitive neuroscience, Dementia

## Abstract

Sharing in embryology and function between the eye and brain has led to interest in whether assessments of the eye reflect brain changes seen in neurodegeneration. We aimed to examine the associations between measures of retinal layer thickness using optical coherence tomography (OCT) and multimodal measures of brain structure and function. Using a convenient sample of twins discordant for type 2 diabetes, we performed cognitive testing, structural brain MRI (tissue volumetry), diffusion tensor imaging (white matter microstructure), and arterial spin labelling (cerebral blood flow). OCT images were recorded and retinal thickness maps generated. We used mixed level modelling to examine the relationship between retinal layer thicknesses and brain measures. We enrolled 35 people (18 pairs, mean age 63.8 years, 63% female). Ganglion cell layer thickness was positively associated with memory, speed, gray matter volume, and altered mean diffusivity. Ganglion cell layer thickness was strongly positively associated with regional cerebral blood flow. We found only a limited number of associations between other retinal layer thickness and measures of brain structure or function. Ganglion cell layer thickness showed consistent associations with a range of brain measures suggesting it may have utility as a marker for future dementia risk.

## Introduction

There is great interest in identifying biomarkers that may indicate a risk of future dementia in people at mid-life and enable research into targeted prevention. Many such biomarkers are based on direct neuroimaging or specimens involving blood or cerebrospinal fluid. These procedures are often complex, expensive, or invasive. The retina, which can be relatively simply visualised, has been postulated as a potential surrogate of brain health given that its vascular and neuronal structures are an extension of that of the brain^1–3^. Imaging of the retina therefore allows direct visualisation of cerebrovascular health as well as neuronal integrity. Links between retinal structure and brain health have been described previously and as technology has evolved, so too has our ability to gain better understanding of potential mechanistic pathways^4^. While the usefulness of retinal vasculature for this purpose is still debated^4–11^, it has been postulated that optical coherence tomography (OCT) may be more attractive by visualising the different layers of the retina at very high resolution, thus providing a direct measure of neuronal integrity in neurodegenerative conditions such as multiple sclerosis and dementia^12–15^. For example, a systematic review and meta-analysis of 17 studies reported that OCT may have some utility in differentiating between Alzheimer’s Disease, Mild Cognitive Impairment and healthy controls^14^.

To date, the use of OCT in dementia research has focussed on associations between OCT and clinical cognitive syndromes as a marker of disease. However, few studies have examined the links between retinal layer thickness and neuroimaging measures of mechanistic pathways potentially involved in the development of dementia. In one study of 164 people without dementia, ganglion cell-inner plexiform layer thickness measured using OCT was associated with global and regional cerebral atrophy^16^ supporting the potential of OCT to act as a marker of neurodegenerative processes. However, these need replication, and it also remains unclear whether retinal sublayer thickness measures may correlate with other brain changes such as cerebral perfusion, white matter tract integrity or cognition.

In this study, we aimed to study the associations between several retinal OCT measures with multimodal neuroimaging markers (including structure and perfusion), and cognitive function, using the extensive measurements collected in a convenient sample of twins recruited for the study of diabetes and brain health.

## Methods

### Sample

The convenient sample used for this study comprised of co-twins with and without type 2 diabetes (T2D) derived from the Australian Twin Registry as part of a parent study aimed at understanding the impact of type 2 diabetes (T2D) on the brain. In that parent study, twin pairs ≥ 50 years old discordant for T2D were recruited using the Australian Twin Registry as a sampling frame^17^. Exclusion criteria were a history of significant neurological disease (seizures, dementia, Parkinson’s Disease, severe head trauma), those with insufficient English language ability for cognitive testing, or contraindication to Magnetic Resonance Imaging (MRI). T2D diagnosis was based on previous physician diagnosis and T2D status confirmed with fasting glucose level ≥ 7.0 mmol/L (≥ 126 mg/dL), and their co-twin having a fasting glucose level of < 7.0 mmol/L (< 126 mg/dL), confirming absence of T2D. The study was approved by the Monash University Human Research Ethics Committee and the Monash Health Human Research Ethics Committee. Informed consent was obtained from all participants and all measurements were performed in accordance with relevant guidelines and regulations.

### Measurements

#### Cognitive measures

Cognitive tests included the Hopkins Verbal Learning Test (verbal memory)^18^ Rey-Osterrieth Complex Figure Test copy (visuospatial, perceptual function, organisation) and delay (visual memory)^18^, components of the Cambridge Neuropsychological Test Automated Test Battery including paired associate learning, (episodic memory and learning), and simple reaction time (speed)^19^, components from the Wechsler Memory Scale 3^rd^ version including mental control (maintaining mental set), digit span forwards (simple attention) and backwards (working memory)^18^. The National Adult Reading Test was conducted to assess premorbid intelligence^20^.

#### Retinal measures

Both eyes of each participant were imaged using the spectral-domain Heidelberg Spectralis OCT (Heidelberg Engineering, Heidelberg, Germany) with Heidelberg Eye Explorer software (HEYEX Version 5.7) after pupil dilation with 1% tropicamide and 2.5% phenylephrine. For macula imaging, a horizontal and vertical 30-degree line scan was performed through the foveal centre with the automatic real time set to average 36 scans. For peripapillary imaging, the default peripapillary OCT nerve fibre layer thickness scan protocol for HEYEX Version 5.7 was performed. The acquired OCT data was then exported into E2E-format files for transmission to the independent masked reading centre.

All OCT images were manually checked at the reading centre to ensure there were no significant retinal sublayer segmentation errors. The macular and peripapillary OCT measurements were performed using the inbuilt Heidelberg Spectralis mapping software. Macular measurements were automatically averaged across each of the Early Treatment of Diabetic Retinopathy Study (ETDRS) segments: the central foveal subfield within the inner 1 mm diameter circle; the inner circle subfield between the inner and middle 3 mm diameter circles; and the outer circle subfield between the middle and outer 6 mm diameter circles. Both the inner and the outer circles were divided into superior, nasal, inferior, and temporal quadrants, creating 9 segments. Peripapillary nerve fibre layer thickness measurements were recorded around the optic nerve dividing it into 6 segments, superior nasal, nasal, inferior nasal, inferior temporal, temporal and then a global average.

Segmentation of the retinal layers was performed on macular volume scans using graph-based, automated methods^21^ within the Heidelberg engineering software. This created the retinal nerve fibre layer, ganglion cell layer, inner plexiform layer, inner nuclear layer, outer plexiform layer, outer nuclear layer, retinal pigment epithelium and total retinal thickness. The averaged thicknesses of these retinal layers in the 9 ETDRS segments were recorded in microns. The Spectralis system sets the outer retinal threshold line as Bruch’s membrane^22^, so the total retinal thickness was measured as the distance from the internal limiting membrane to Bruch’s membrane.

Retinal photographs were manually reviewed, and diabetic retinopathy graded based on the Airlie House protocol by a trained expert (CC).

#### Brain MRI

MRI data were obtained using a 3 T Siemens Magnetom Skyra scanner (Siemens, Erlangen, Germany). The parameters used for T1 acquisition were: T1 : repetition time (TR) = 1900 ms, echo time (TE) = 2.43 ms, flip angle = 9°, field of view (FOV) = 240 mm, 192 × 192 matrix, and slice thickness = 0.6 mm; Fluid attenuated inversion recovery (FLAIR)—TR = 9000 ms, TE = 81 ms, flip angle = 150°, FOV = 220 mm, acquisition matrix 320 × 217, and slice thickness = 4 mm; Diffusion Tensor Imaging: 64 directions, gradient = 3000 s/mm2, with 1 b = 0 s/mm2 reference image. TR = 15900 ms, TE = 111 ms, FOV = 256 mm, echo spacing = 0.66 ms, Acquisition matrix = 128 × 128, voxel size 2 × 2 × 2 mm; Susceptibility Weighted Imaging: TR = 28 ms, TE = 20.0 ms, FOV = 220 mm, Flip angle = 15, acquisition matrix 384 × 269, slice thickness = 1.2.

### Imaging analysis

#### Derivation of structural measures

FreeSurfer 5.3 was used for initial tissue classification using FLAIR images. The steps involved the following—removing non-brain tissue using a hybrid deformation procedure^23^, automated Talairach transformation, segmentation of the subcortical white matter and deep gray matter volumetric structures^24^, intensity normalization^25^, tessellation of the gray matter white matter boundary, automated topology correction^26,27^, and surface deformation following intensity gradients to optimally place the gray/white and gray/cerebrospinal fluid borders at the location of the greatest shift in signal intensity^28–30^. Hypointensities on T1-weighted scans, corresponding to white matter hyperintensities on FLAIR scans, can cause segmentation errors in FreeSurfer. Therefore, we then conducted automated white matter hyperintensity segmentation on FLAIR using the white matter mask created by FreeSurfer. Intra-subject co-registration was performed between these final segmented FLAIR images against T1-weighted images to generate final tissue classification using Statistical Parametric Mapping 12 software, Wellcome Trust Centre for Neuroimaging. Based on this, structural MRI measures of total brain volume, regional gray matter volumes, and cortical thickness were derived.

Measures of white matter microstructure were derived from diffusion images which were first corrected for movement and eddy current distortion using the Functional MRI Brain Software Library “eddy” tool. Images for fractional anisotropy (FA), mean diffusivity (MD), axial diffusivity and radial diffusivity were created using the Functional MRI Brain Software Library tool, *dtifit* which fits a diffusion tensor model at each voxel. The tract-based spatial statistics pipeline^31^ was used to align all participant diffusion data with a common space and project data onto an FA skeleton. A FreeSurfer lobe parcellation of the 1 mm Montreal Neurological Institute template was used to compute mean FA, MD, and axial and radial diffusivity per lobe.

#### Cerebrovascular lesions

The presence of cerebral infarcts and cerebral microbleeds was determined by visual identification and consensus between two trained experts (CK, VS), as published previously^32^. White matter hyperintensity segmentation was carried out using the automated segmentation method described in our previous work^33^ and the FreeSurfer white matter mask.

#### Cerebral perfusion

Arterial Spin Labelling and FLAIR images were first aligned with the T1-weighted images using the co-registration facility in the Statistical Parametric Mapping 12 software, Wellcome Trust Centre for Neuroimaging. Cerebral Blood Flow (ml/min/100 g brain tissue) was estimated from the Arterial Spin Labelling images using Bayesian Inference for Arterial Spin Labelling^34^ from the Functional MRI Brain Software Library. A nonlinear transformation to standard space was estimated using the Statistical Parametric Mapping 12 unified segmentation procedure applied to the T1-weighted images, and used to transform cerebral blood flow images to standard space for voxel-wise statistics. A lobe-based parcellation was also produced and mean cerebral blood flow was computed for each lobe using the FreeSurfer output^35^.

#### Other measurements

Demographic information and medical history including risk factors for cardiovascular disease, complications of diabetes, use of medications, and age of diabetes onset were recorded using a standardized, structured questionnaire. Participants were asked to report whether they had been diagnosed by a physician with hypertension, hyperlipidaemia, stroke or coronary artery disease. Height, weight, waist and hip circumference were measured and used to calculate body mass index and waist-hip ratio. Two readings were taken for each measurement, and if there was a difference of 2 cm or more between these two readings, a third was performed, and the average of the measurements was used. Blood samples were analysed for fasting glucose, Hb_A1c_, and insulin levels. A Beckman Coulter DXC800 Analyser was used to determine glucose concentration using oxygen rate method utilising a glucose oxygen electrode (Beckman Coulter, Inc, Indianapolis, IN, USA) and Hb_A1c_ was measured using an ADAMS ARKRAY Glycohaemoglobin Analyser HA8160 (ARKRAY, Inc, Kyoto, Japan). Serum insulin levels were measured using the immune-enzymatic Access/DXI Ultrasensitive Insulin assay (Beckman Coulter, Inc, Indianapolis, IN, USA) and insulin resistance was calculated using the Homeostatic Model Assessment method^36^.

### Statistical analyses

Using the “factor…varimax” commands within Stata statistical package software (version 14.0, StataCorp, College Station, TX)^38^ neuropsychological test scores were subjected to data reduction using principal component factor analysis with orthogonal rotation, yielding three factors. Based on loading of tests, the factors represented the cognitive domains of attention, memory, and perceptual speed.

**Figure 1:**
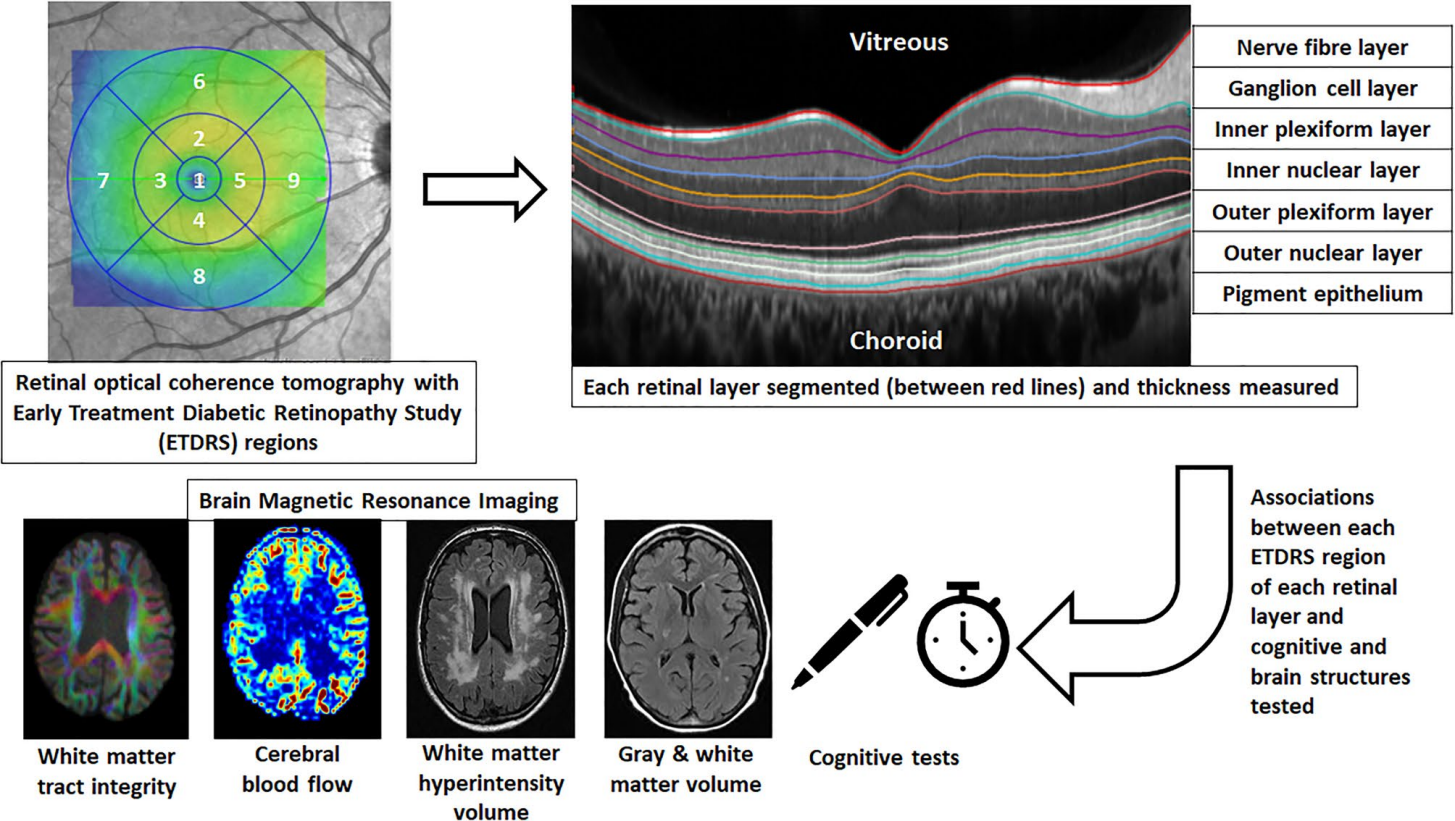
Work design scheme summarizing the overall analytical approach with examples of the retinal and brain measurements.

We used Stata version 14.0 for all analyses. Linear mixed modelling was used to examine the associations between each of the ETDRS regions and the brain outcome of interest adjusting for age, sex and T2D. Models of cognitive outcomes included additional adjustment for education. Twin number was entered as a random effect, so that the model allowed partitioning of within-pair and between-pair variance. To reduce the risk of false positives from multiple comparisons (right and left eye segments compared with three cognitive and six brain structural outcomes i.e., 18 independent tests of association), we used Bonferroni correction (0.05/18 = *p* < 0.002) across each of the cognitive and imaging modalities. Figure 1 presents a work design scheme summarizing the overall analytical approach with examples of the retinal and brain measurements.

## Results

### Sample

Of the 90 twin pairs approached between April 2015 and May 2016, there were 10 pairs in which at least one twin did not respond, 45 pairs in which at least one twin declined to participate, and 9 pairs who agreed to participate but were unable to meet the logistical requirements to attend all study assessments. A total of 18/90 pairs were enrolled. Participant characteristics summarized by diabetes status are presented in Table 1. The mean age of participants was 63.8 years (SD 6.7) and those with T2D had a mean duration of disease of 8.2 years (SD 5.2) and HbA_1c_ 7.0% (SD 1.2%). There were 5 individuals without T2D that had fasting glucose concentrations greater than 5.5 mmol/L consistent with pre-diabetes^40^. The mean diabetic retinopathy score was 12.5 (SD 5.23) with the poorest score for the presence of microaneurysm. Supplementary Tables 1–3 show the various retinal sublayer thicknesses within each of the ETDRS segments for each eye, showing that twins with and without T2D had similar retinal sublayer thicknesses in both eyes.

**Table 1:** Characteristics of participants.

	Type 2 diabetes *n* (%) or mean (SD) (*N* = 17)	No Type 2 diabetes *n* (%) or mean (SD) (*N* = 18)
Age (years)	63.8 (6.7)	63.7 (6.7)
Female sex	10 (59)	12 (67)
Attained college degree	5 (30)	6 (33)
Geriatric depression scale score	5.3 (2.0)	6.0 (3.1)
Hypertension	13 (76)	10 (56)
Defined daily dose of antihypertensive medication	1.89 (1.52)	0.85 (1.11)
Hyperlipidaemia	13 (76)	8 (44)
Stroke	1 (6)	0 (0)
Myocardial infarction	2 (12)	0 (0)
Ever smoker	9 (53)	8 (44)
National adult reading test intelligence quotient	108 (12.4)	110 (8.9)
Systolic blood pressure (mmHg)	126 (10)	125 (11)
Diastolic blood pressure (mmHg)	74 (7.7)	78 (6.7)
Body mass index	34.8 (6.8)	29.4 (6.1)
Fasting glucose (mmol/L)	7.7 (1.8)	5.3 (0.5)
Haemoglobin A1c (%)	7.0 (1.2)	5.6 (0.3)
Duration of type 2 diabetes (years)	8.2 (5.2)	N/A
Homeostatic model assessment 2 (mg/dL)	1.7 (1.3)	1.1 (1.5)
Diabetic retinopathy grading	13.6 (7.3)	10.6 (2.4)

### Associations between retinal sublayer thickness and brain measures

**Figure 2:**
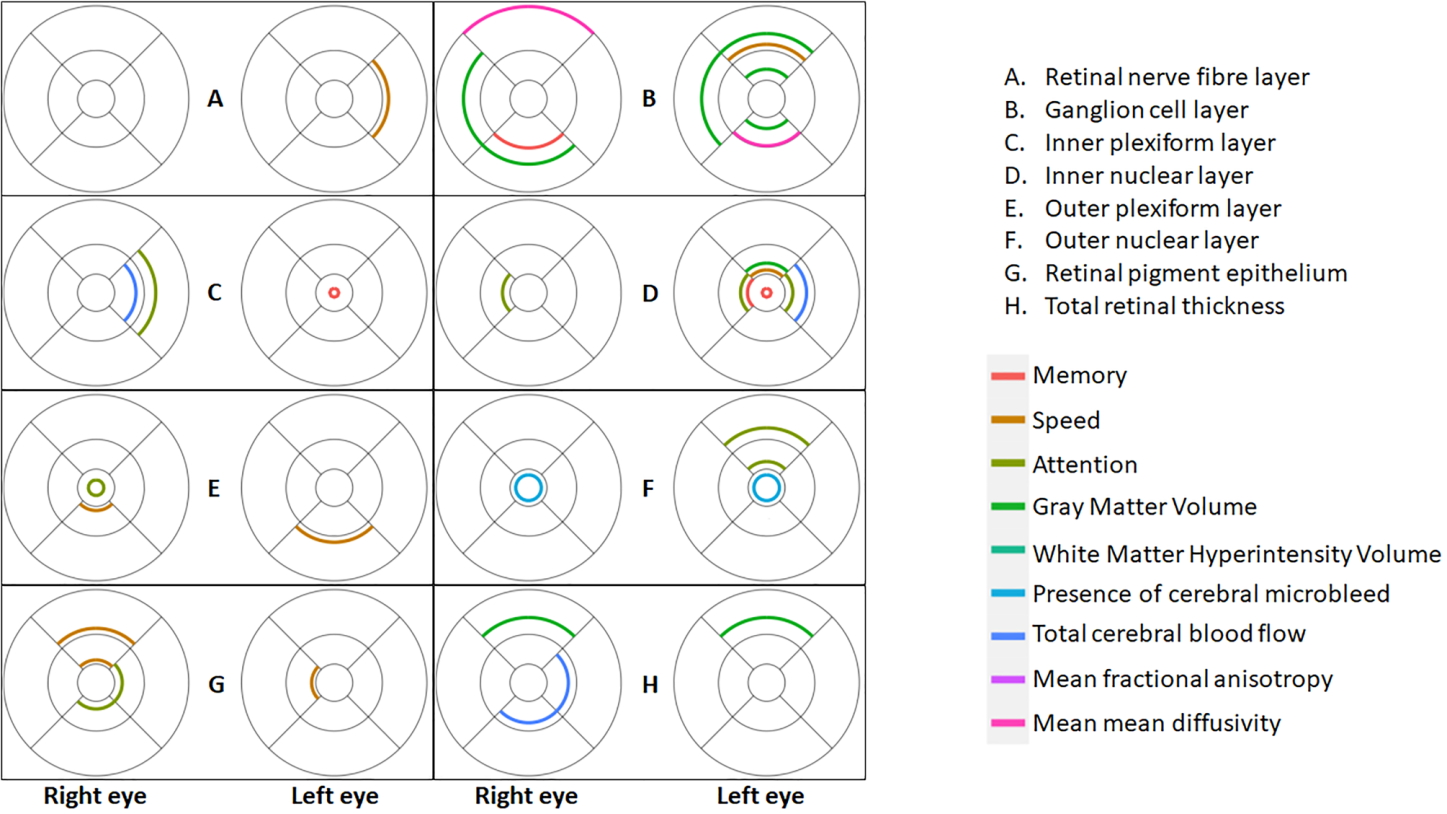
Positive associations between each retinal layer by Early Treatment of Diabetic Retinopathy Study segment and brain outcomes.

We explored the associations of the individual retinal sublayer thickness measures with cognitive function and brain MRI measures, by ETDRS segments, corrected for multiple comparisons, adjusted for age, sex and T2D (Supplementary Tables 4–11). The positive associations between each retinal layer by ETDRS segment are summarized in Fig. 2.

Retinal nerve fibre layer thickness per se was not associated with cognitive or MRI measures (Supplementary Table 4), but ganglion cell layer thickness was positively associated with memory (segment 8, right eye), speed (segment 6, left eye), and strongly in both eyes with gray matter volume (all *p* < 0.002) (Supplementary Table 5). In both eyes, ganglion cell was also associated with altered mean diffusivity (segment 6, right eye and segment 4, left eye). In secondary analyses, we also found that ganglion cell layer thickness in both eyes and in many segments was strongly positively associated with regional cerebral blood flow, particularly in the insula and temporal lobes (Table 2). We found a limited number of associations between synaptic layers (inner plexiform and outer plexiform) and brain measures (Supplementary Tables 6 and 8). We found associations between inner nuclear layer thickness (Supplementary Table 7) and memory (segments 1 and 5 left eye), speed (segment 2, left eye), attention (segment 3, both eyes, segment 5 left eye), gray matter (segment 2, left eye), total cerebral blood flow (segment 3, left eye). There were associations between outer nuclear layer thickness (Supplementary Table 9) and attention (segment 6, left eye) and cerebral microbleeds (segment 1, both eyes). We found associations between retinal pigment epithelium layer thickness (Supplementary Table 10) and speed (segments 2 and 6 right eye and segment 5 left eye) and attention (segments 4 and 5 right eye). Total retinal thickness was not associated with cognitive scores but was positively associated with greater gray matter volume (segment 6, both eyes), and greater total cerebral blood flow (segment 4, 5, right eye) (Supplementary Table 11).

**Table 2:** Associations between regional Ganglion Cell layer thickness and regional cerebral blood flow.

	Thickness of early treatment of diabetic retinopathy study segment (µm)	Right occipital β	Left occipital β	Right frontal β	Left frontal β	Right parietal β	Left parietal β	Right insula β	Left insula β	Right temporal β	Left temporal β
Right eye	1	− 0.32	0.99	0.27	0.61	0.18	0.82	0.13	0.52	− 0.04	0.62
	2	0.73	1.13*	0.64*	0.82*	0.56	0.86*	1.14*	1.35*	0.73*	1.25*
	3	0.31	0.76	0.57*	0.77*	0.46	0.83*	1.04*	1.11*	0.56	0.91*
	4	0.24	0.61	0.56*	0.67*	0.47	0.72*	1.15*	1.09*	0.59	0.91*
	5	0.53	0.94	0.74*	0.84*	0.68*	0.89*	1.25*	1.24*	0.72*	1.06*
	6	0.31	0.46	0.01	− 0.14	0.11	0.003	0.55	0.44	0.16	0.52
	7	1.06	1.18	0.36	0.41	0.40	0.70	0.75	1.09	0.33	1.00
	8	1.67	1.50*	0.41	0.47	0.83*	0.85	1.24*	1.42*	0.99	1.37*
	9	0.53	0.83	0.38	0.40	0.36	0.45	1.05*	1.11*	0.60	1.07*
Left eye	1	− 0.18	0.18	0.18	0.49	0.28	0.60	0.15	0.42	0.06	0.40
	2	0.56	0.88	0.55*	0.64*	0.44	0.67*	1.07*	1.15*	0.61	0.95*
	3	0.56	0.90	0.56*	0.56*	0.62*	0.73*	1.07*	1.04*	0.66*	0.91*
	4	0.61	0.84	0.49	0.54	0.46	0.63	1.16*	1.17*	0.62	0.97*
	5	0.48	0.57	0.43	0.68*	0.36	0.68*	0.74*	0.96*	0.37	0.67
	6	1.06	1.36	0.40	0.53	0.54	0.68	1.07*	1.28*	0.42	1.11
	7	0.82	0.77	0.57	0.51	0.70	0.52	1.23*	1.09*	0.84	1.15*
	8	1.45	1.24	0.72	0.48	0.80	0.53	1.25*	1.16	1.06	1.17
	9	0.61	1.17	0.39	0.44	0.30	0.59	0.90	1.18*	0.31	1.01

Adjusted for age, sex, type 2 diabetes and, where appropriate, total intracranial volume *p < 0.002 with Bonferroni correction for multiple comparison.

## Discussion

In this convenient sample of twins, we found that individual retinal layer thicknesses were variably associated with brain structure and function. In particular, we found that ganglion cell layer (GCL) thickness was most likely to be associated with a range of brain measures including cognition, gray matter volume, white matter microstructure and cerebral blood flow, after adjustments and correction for multiple comparisons, suggesting its utility as a marker for future risk of neurodegeneration and dementia.

The GCL is composed of a high density of neurons that are primarily responsible for transmission of visual stimuli to the cortex and are highly energy dependent^41^, potentially explaining the higher likelihood of GCL being associated with measures of cortical structure and brain function. Changes in the GCL have also been shown to be associated with Alzheimer’s Dementia (AD) and neurodegeneration in a number of studies^2,12–14,42,43^. In early work performed in a Chinese sample of people with normal cognition, Mild Cognitive Impairment (MCI) and AD, the authors reported that GCL thickness was lower in those with either AD or MCI than controls^2^. In this study, the authors postulated that the loss of the ganglion cell bodies that make up the GCL was an early marker of cerebral neurodegeneration seen in early AD. These results have been supported by the results of subsequent metanalyses that have included between 11 and 17 observational studies comparing people aged from 60 to 80 years with dementia (approximately 360 people) or Mild Cognitive Impairment to cognitively healthy controls (approximately 350 people)^12,14^. Although none of the participants in our study had a diagnosis of dementia, our results suggest that the results of previous work can be extended and that GCL thickness may be a marker of subtle brain changes also seen in neurodegenerative disease.

The mechanisms underlying the associations between GCL thickness and neurodegeneration remain unclear. The strong associations between GCL thickness and regional cerebral blood flow in our study raises speculation whether reduced vascular supply, AD pathology (e.g., amyloid [Aβ] and tau) or both may be involved in causing loss of ganglion cells. In a post-mortem, human study, Aβ was found inside and around the melanopsin subtype of ganglion cells suggesting this region is particularly vulnerable to AD processes^44^. AD has also been associated with vascular disease with previous work reporting an association between AD and narrowing of retinal vessels and reductions in retinal venous blood flow rate^45,46^. In a previous study of a single twin pair discordant for AD, the authors reported that AD was associated with reductions in retinal blood flow and vessel diameter^47^. It is possible that the binding of Aβ to vascular endothelial growth factor and Aβ confinement within plaques and accumulation of Aβ deposits in internal vessel walls leads to occlusion of vascular structures and reduced blood flow in both the retina and in the brain^45,48^. Other conditions closely related to dementia may also provide important mechanistic insights^49,50^. Glaucoma commonly co-exists with dementia and both conditions appear to share Apolipoprotein E genetic risk factors and a predilection for loss of retinal ganglion cells^49,50^. Recent research has also reported that mechanisms seen in dementia such as Aβ, Tau and insulin receptor pathways may all play important roles in the ganglion cell loss seen in glaucoma and that these pathways may be similar or overlap with those seen in AD^49,50^. Glaucoma treatment strategies may also provide some important insights to potential mechanisms. The mainstay of glaucoma treatment is lowering of intraocular pressure. However, a sub-group of people with glaucoma have normal intraocular pressure and benefit less from lowering of intraocular pressure. Although it remains unclear whether people with normal tension glaucoma have a higher risk of dementia than those with elevated intraocular pressure, vascular dysregulation appears to be more pronounced in people with normal tension glaucoma than those with elevated intraocular pressure^49^. The mechanisms behind the ganglion cell loss in this condition appears to be more closely related to reperfusion injury than ischaemia and has led some clinicians to use nocturnal blood pressure lowering agents in attempt to reduce variability of perfusion^49^. It is unclear if altered perfusion is driving any of the neurodegenerative changes we report, but it highlights the potential benefits of understanding the potential pathophysiological overlaps between the eye and the brain. Although there has been little research exploring this relationship to date, this potential link is beginning to be recognized as an important research question^51^.

We did not find a large number of associations between the other retinal layers and brain measurements. The correlation between retinal histology and OCT is greater in the inner retinal layers than the outer layers ^52^. This difference in the sensitivity of OCT to histological change may therefore make us more likely to identify subtle changes in the inner retinal layers than outer layers. Other reasons for the smaller number of associations we see found in outer retinal regions may be due to our small sample size and a low burden of retinopathy ^53^.

Our study has strengths and weaknesses. Strengths include the use of comprehensive measures of retinal health, cognition and structural imaging thus allowing a more sensitive and precise way to identify subtle effects at late midlife that is not limited to single brain measure. Our sample was convenient and borrowed from a study originally intended to study the impact of T2D. As such, the generalisability of our results is unclear. However, our use of mixed level modelling to partition the variance between and within twins reduces the potential of T2D to confound our results.

In summary, retinal ganglion cell layer thickness was found to be associated with measures of cognition, brain structure and blood flow in a sample of well phenotyped middle aged twins. This raises possibilities about both its suitability as an additional biomarker of future risk of neurodegeneration and dementia, as well as their potential underlying mechanisms.

## Supplementary Information

Supplementary Information.

## Data Availability

The datasets analysed during the current study are not publicly available due to participant privacy, but may be available from the corresponding author on reasonable request.
