# Author Correction: Neuroimaging and cognitive correlates of retinal Optical Coherence Tomography (OCT) measures at late middle age in a twin sample

**DOI:** 10.1038/s41598-022-15446-6

**Published:** 2022-06-27

**Authors:** Chris Moran, Zheng Yang Xu, Hemal Mehta, Mark Gillies, Chris Karayiannis, Richard Beare, Christine Chen, Velandai Srikanth

**Affiliations:** 1 National Centre for Healthy Ageing, Melbourne, Australia; 2 Department of Geriatric Medicine, Peninsula Health and Central Clinical School, Monash University, Melbourne, Australia; 3 Department of Aged Care, Alfred Health, Melbourne, Australia; 4 Royal Free London NHS Foundation Trust, London, UK; 5 UCL Medical School, London, UK; 6 Macular Research Group, University of Sydney, Sydney, Australia; 7 Department of Ophthalmology, Monash Health, Melbourne, Australia

Correction to: *Scientific Reports* 10.1038/s41598-022-13662-8, published online 10 June 2022

The Funding section in the original version of this Article was incomplete.

"NHMRC (National Health and Medical Research Council) project grant (application ID 1063608). This project received seed funding from a Pfizer Neuroscience Research Grant (2011) (application ID WS1931543). Chris Moran was supported by a NHMRC-ARC (Australian Research Council) Dementia Research Fellowship (application ID 1109482)."

now reads:

“NHMRC (National Health and Medical Research Council) project grant (application ID 1063608). This project received seed funding from a Pfizer Neuroscience Research Grant (2011) (application ID WS1931543). Chris Moran was supported by a NHMRC-ARC (Australian Research Council) Dementia Research Fellowship (application ID 1109482). The Australian Twin Registry (now Twins Research Australia) receives support from the National Health and Medical Research Council through a Centre of Research Excellence Grant, which is administered by the University of Melbourne.”

The original Article has been corrected.

