# Supplementary material for: Neuroimaging and cognitive correlates of retinal Optical Coherence Tomography (OCT) measures at late middle age in a twin sample
Source: Sci Rep. 2022 Jun 10;12:9562. doi: 10.1038/s41598-022-13662-8 (PMC9187769; doi:10.1038/s41598-022-13662-8)
Supplement: Supplementary file 1 — Supplementary Information. [file 41598_2022_13662_MOESM1_ESM.pdf]

Neuroimaging and cognitive correlates of Retinal Optical Coherence Tomography (OCT) measures at late middle age in a twin sample

ǂChris Moran[1,2,3], ǂZheng Yang Xu[4,5], Hemal Mehta[4,6], Mark Gillies[6], Chris Karayiannis[1,2], Richard Beare[1,2], Christine Chen[7], *Velandai Srikanth[1,2]

Supplementary Table 1 Retinal layer thicknesses in those with and without type 2 diabetes

**Total retinal thickness**				
	Right eye	Right eye	Left eye	Left eye
	No T2D	T2D	No T2D	T2D
ETDRS segment	Mean Thickness (sd) (µm)	Mean Thickness (sd) (µm)	Mean Thickness (sd) (µm)	Mean Thickness (sd) (µm)
1	279.1 (19.2)	281.1 (23.5)	280.1 (17.7)	297.1 (77.0)
2	337.8 (20.5)	337.2 (20.0)	337.7 (19.7)	357.8 (95.0)
3	338.8 (19.0)	338.1 (19.9)	325.0 (19.5)	341.6 (78.9)
4	333.2 (19.3)	331.8 (19.8)	333.5 (20.1)	334.3 (21.8)
5	325.5 (18.0)	325.2 (18.9)	339.7 (18.8)	347.3 (47.2)
6	289.5 (14.2)	290.7 (16.3)	292.8 (14.4)	290.5 (16.2)
7	308.1 (14.6)	305.3 (16.9)	282.1 (15.1)	278.8 (16.2)
8	280.4 (12.8)	287.1 (23.0)	280.1 (14.1)	291.5 (60.9)
9	280.7 (14.7)	281.0 (15.6)	308.8 (17.0)	304.9 (18.7)
**Retinal Nerve Fibre Layer**				
1	13.0 (2.52)	12.7 (2.6)	13.4 (2.2)	27.2 (64.3)
2	23.5 (3.2)	24.5 (3.5)	24.7 (5.0)	44.9 (95.2)
3	19.7 (3.0)	20.7 (2.8)	17.7 (2.1)	34.5 (72.7)
4	22.7 (3.0)	23.9 (5.3)	24.0 (2.9)	24.4 (3.1)
5	17.3 (1.1)	19.2 (4.0)	20.9 (3.6)	29.1 (39.4)
6	34.1 (5.0)	37.3 (5.7)	35.9 (6.8)	36.4 (7.3)
7	43.7 (9.6)	45.7 (8.4)	19.2 (2.3)	19.4 (2.5)
8	35.2 (6.9)	38.5 (13.7)	35.8 (5.1)	44.7 (38.0)
9	18.9 (1.4)	19.9 (2.7)	44.7 (8.0)	45.1 (9.5)
**Retinal Ganglion Cell Layer**				
1	15.1 (2.8)	15.9 (3.5)	16.5 (5.7)	16.4 (5.1)
2	49.5 (6.0)	49.8 (6.6)	49.1 (5.4)	49.4 (7.1)
3	48.4 (6.3)	48.4 (7.0)	43.2 (5.9)	42.6 (7.3)
4	48.6 (6.1)	47.7 (6.6)	48.8 (5.3)	48.5 (6.3)
5	42.9 (5.9)	43.1 (6.3)	48.7 (4.9)	47.3 (7.2)
6	32.3 (3.0)	31.8 (4.3)	32.4 (3.4)	32.3 (4.0)
7	36.8 (3.1)	36.2 (4.3)	33.6 (4.5)	32.5 (4.6)
8	31.6 (2.5)	31.2 (4.7)	31.9 (3.1)	31.3 (3.4)
9	32.9 (4.6)	33.3 (4.9)	36.9 (3.3)	35.6 (4.5)

Supplementary Table 2 Retinal layer thicknesses in those with and without type 2 diabetes

**Inner Plexiform Layer**				
	Right eye	Right eye	Left eye	Left eye
	No T2D	T2D	No T2D	T2D
ETDRS segment	Mean Thickness (sd) (μm)	Mean Thickness (sd) (μm)	Mean Thickness (sd) (μm)	Mean Thickness (sd) (μm)
1	21.4 (2.8)	21.6 (3.2)	22.0 (3.7)	22.7 (5.7)
2	40.2 (4.2)	39.5 (4.7)	40.0 (3.9)	40.2 (4.8)
3	40.9 (4..3)	40.7 (4.5)	40.0 (4.1)	40.2 (4.9)
4	39.5 (4.3)	39.2 (4.8)	39.7 (4.2)	39.4 (4.8)
5	40.1 (4.2)	398 (4.1)	41.2 (3.5)	40.4 (4.8)
6	26.8 (3.0)	26.5 (3.0)	26.6 (2.8)	26.6 (2.7)
7	28.8 (3.0)	27.8 (3.3)	31.6 (3.0)	31.4 (3.5)
8	26.4 (2.7)	25.9 (3.0)	26.2 (2.4)	26.1 (3.1)
9	30.9 (3.0)	31.6 (3.1)	28.4 (2.5)	27.7 (3.5)
**Inner Nuclear layer**				
1	21.0 (4.3)	21.3 (5.0)	21.5 (4.6)	22.8 (7.5)
2	40.5 (3.8)	40.6 (4.5)	40.8 (4.7)	40.8 (5.6)
3	40.7 (3.5)	41.0 (3.9)	37.6 (4.7)	38.5 (4.3)
4	41.2 (3.6)	40.3 (3.9)	39.9 (4.0)	40.0 (3.7)
5	39.2 (2.7)	38.0 (3.8)	41.3 (4.2)	41.5 (4.2)
6	30.6 (2.7)	30.7 (3.2)	31.8 (3.3)	31.0 (3.1)
7	33.6 (2.6)	33.1 (3.2)	33.3 (3.5)	32.5 (2.8)
8	31.2 (2.5)	31.3 (2.4)	30.8 (2.7)	31.2 (3.5)
9	33.4 (3.2)	33.2 (3.1)	34.2 (2.8)	33.4 (3.3)
**Outer Plexiform layer**				
1	26.1 (5.8)	27.2 (5.2)	26.1 (4.7)	27.1 (4.7)
2	30.4 (7.1)	34.3 (11.9)	32.4 (8.1)	38.3 (13.1)
3	31.1 (2.8)	33.4 (6.7)	32.2 (4.9)	34.7 (5.6)
4	39.1 (12.7)	36.2 (15.6)	33.6 (8.1)	34.4 (20.7
5	33.3 (6.8)	32.7 (5.7)	30.5 (3.4)	34.0 (7.6)
6	35.4 (45.7)	26.7 (4.0)	25.3 (1.7)	27.3 (3.9)
7	27.8 (2.1)	29.0 (3.1)	27.1 (1.3)	28.3 (3.4)
8	27.7 (3.8)	26.4 (3.6)	27.3 (3.7)	26.0 (3.3)
9	27.6 (2.5)	27.3 (3.3)	28.1 (3.3)	28.8 (3.5)

Supplementary Table 3 Retinal layer thicknesses in those with and without type 2 diabetes

**Outer Nuclear layer**				
	Right eye	Right eye	Left eye	Left eye
	No T2D	T2D	No T2D	T2D
ETDRS segment	Mean Thickness (sd) (µm)	Mean Thickness (sd) (µm)	Mean Thickness (sd) (µm)	Mean Thickness (sd) (µm)
1	95.8 (15.7)	96.6 (8.4)	95.3 (13.6)	94.8 (10.9)
2	71.6 (12.5)	67.5 (13.9)	69.1 (11.1)	64.3 (13.7)
3	75.3 (11.3)	72.6 (11.9)	72.3 (10.9)	69.5 (11.1)
4	61.6 (14.8)	64.5 (15.1)	67.5 (12.6)	67.9 (13.9)
5	70.3 (10.9)	72.2 (10.4)	74.8 (11.1)	72.6 (12.1)
6	61.1 (8.8)	60.0 (8.9)	60.9 (8.4)	58.1 (7.0)
7	57.6 (9.3)	55.5 (8.6)	58.3 (8.0)	56.6 (9.1)
8	50.8 (9.2)	55.8 (22.3)	50.8 (10.5)	53.5 (12.6)
9	57.1 (7.4)	57.1 (7.5)	57.5 (9.4)	56.5 (10.2)
**Retinal Pigment Epithelium**				
1	16.3 (1.7)	15.6 (3.6)	15.8 (2.5)	15.2 (1.8)
2	15.1 (1.5)	14.5 (1.5)	15.2 (1.7)	18.8 (19.0)
3	15.4 (2.2)	14.4 (1.7)	14.5 (1.2)	14.3 (2.3)
4	14.5 (1.8)	14.1 (1.9)	14.5 (1.8)	14.1 (1.4)
5	14.6 (1.6)	14.1 (1.2)	15.0 (1.9)	14.8 (1.5)
6	13.7 (1.8)	13.2 (1.1)	13.7 (1.9)	13.8 (2.2)
7	13.6 (1.8)	12.8 (1.2)	13.1 (1.1)	12.8 (1.3)
8	12.9 (1.5)	13.6 (3.7)	13.1 (1.6)	14.7 (9.1)
9	13.2 (1.3)	13.0 (0.9)	13.6 (1.6)	13.0 (1.1)
**Peripapillary retinal nerve fibre layer**				
Global	92.8 (13.8)	96.3 (14.2)	92.7 (15.7)	94.7 (15.1)
Superior nasal	84.3 (28.9)	94.2 (24.1)	95.2 (31.7)	96.0 (30.2)
Nasal	67.7 (18.5)	70.9 (20.1)	67.7 (20.5)	72.1 (21.9)
Inferior nasal	100.0 (22.9)	109.8 (31.3)	102.5 (32.3)	106.8 (27.0)
Inferior temporal	145.8 (23.5)	141.7 (22.8)	139.9 (22.3)	139.2 (29.9)
Temporal	73.9 (15.5)	76.0 (15.2)	71.4 (16.2)	71.7 (17.5)
Superior temporal	129.4 (25.1)	131.7 (23.6)	126.0 (33.9)	127.7 (26.7)

Supplementary Table 4 Retinal Nerve Fibre Layer Thickness – associations with cognition, brain structure, global cerebral blood flow and glucose uptake

Thickness of Early Treatment of Diabetic Retinopathy Study segment (μm)	Memory β	Speed β	Attention β	Gray Matter Volume (ml) β	White Matter Hyperintensity Volume (ml) β	Presence of Cerebral Microbleed β	Total Cerebral Blood Flow (ml/min /100g) β	Mean Fractional Anisotropy β	Mean Mean Diffusivity (mmx10^{-7}) β
Right eye									
1	-0.10	-0.009	-0.04	-992	-33.2	0.009	-0.02	3.3 x10^{-4}	-1.5 x10^{-6}
2	0.008	-0.06	-0.02	570	-130.0	-0.021	1.46	-0.001	-1.56 x10^{-7}
3	-0.04	-0.09	-0.04	-142	92.7	-0.002	0.70	-0.001	-1.7 x10^{-6}
4	0.01	0.04	0.008	2232	72.1	6.8 x10^{-4}	0.42	-2.2 x10^{-4}	-1.2 x10^{-4}
5	0.03	0.005	-0.05	372	-27.9	0.010	0.50	-2.4 x10^{-4}	-1.1 x10^{-6}
6	-0.01	-0.01	0.03	772	-182.1	-0.01	0.82	1.3 x10^{-4}	-5.6 x10^{-7}
7	-0.004	-0.006	0.003	879	24.1	-2.5 x10^{-5}	0.50	-3.3 x10^{-4}	-3.4 x10^{-7}
8	0.01	0.007	-0.002	919	10.2	-4.8 x10^{-4}	0.39	4.0 x10^{-5}	-3.6 x10^{-7}
9	-0.06	-0.05	-0.003	2803	-42.3	-0.004	1.74	2.6 x10^{-4}	-1.2 x10^{-6}
Left eye									
1	-0.007	3.5 x10^{-4}	0.003	-89	-18.9	-6.4 x10^{-4}	-0.004	1.6 x10^{-5}	-1.9 x10^{-8}
2	-0.004	9.5 x10^{-5}	0.002	-58	-12.7	-4.6 x10^{-4}	5.5 x10^{-4}	7.4 x10^{-6}	-9.8 x10^{-9}
3	-0.006	3.3 x10^{-4}	0.002	-73	-16.2	-5.3 x10^{-4}	-0.002	1.6 x10^{-5}	-1.7 x10^{-8}
4	-0.008	0.004	0.005	2409	-181	-0.10	1.32	0.002	-1.3 x10^{-6}
5	-0.01	3.1 x10^{-5}	0.005	-146	-30.4	-8.8 x10^{-4}	0.006	1.0 x10^{-5}	-2.2 x10^{-8}
6	-0.02	-0.01	-0.004	1536	-111	-0.006	0.67	-1.3 x10^{-4}	2.2 x10^{-7}
7	-0.07	-0.13*	-0.04	507	-10.7	-1.8 x10^{-4}	1.53	-0.001	9.7 x10^{-7}
8	-0.01	5.0 x10^{-4}	0.004	-87	-35.4	-0.002	0.03	6.5 x10^{-5}	-5.5 x10^{-8}
9	-0.006	-0.007	0.003	974	-6.4	-5.3 x10^{-4}	0.47	-3.9 x10^{-4}	-1.1 x10^{-7}

Adjusted for age, sex, type 2 diabetes and, where appropriate, total intracranial volume *$p<0.002$ with Bonferroni correction for multiple comparisons

Supplementary Table 5 Ganglion Cell Layer Thickness – associations with cognition, brain structure, global cerebral blood flow and glucose uptake

Thickness of Early Treatment of Diabetic Retinopathy Study segment (µm)	Memory β	Speed β	Attention β	Gray Matter Volume (ml) β	White Matter Hyperintensity Volume (ml) β	Presence of Cerebral Microbleed β	Total Cerebral Blood Flow (ml/min/100g) β	Mean Fractional Anisotropy β	Mean Mean Diffusivity (mmx10^{-7}) β
Right eye									
1	-0.07	-0.009	-0.009	-1619	-88	-0.006	0.41	9.5 x10^{-5}	-4.9 x10^{-7}
2	0.03	0.03	-0.003	1925	-121	-0.002	0.86	-1.0 x10^{-4}	-5.4 x10^{-7}
3	-0.01	0.02	0.02	1505	-123	-0.004	0.72	2.0 x10^{-4}	-9.0 x10^{-7}
4	0.004	0.02	0.01	1294	-114	-0.007	0.69	-1.1 x10^{-4}	-5.0 x10^{-7}
5	-8.2 x10^{-4}	0.02	0.02	1353	-150	-0.01	0.84	7.2 x10^{-5}	-5.9 x10^{-7}
6	0.04	0.06	0.05	2825	-446	0.001	0.13	0.001	-2.0 x10^{-6}*
7	0.09	0.08	0.03	3651*	-433	-0.005	0.57	0.001	-6.3 x10^{-7}
8	0.13*	0.07	0.03	3137*	-194	-0.008	0.85	8.5 x10^{-4}	-2.1 x10^{-7}
9	0.03	0.03	0.04	1694	-227	-0.007	0.58	5.2 x10^{-4}	-6.1 x10^{-7}
Left eye									
1	-0.06	-0.01	-0.04	-1525	-70	-0.003	-0.31	-7.7 x10^{-4}	5.2 x10^{-7}
2	0.02	0.03	0.02	2032*	-158	-0.005	0.71	2.9 x10^{-4}	-9.1 x10^{-7}
3	0.006	0.02	0.01	1521	-177	-0.01	0.71	6.9 x10^{-4}	-8.1 x10^{-7}
4	0.02	0.04	0.01	2231*	-192	-0.008	0.70	7.2 x10^{-4}	-1.3 x10^{-6}*
5	0.03	0.02	0.006	1916	-100	-8.5 x10^{-4}	0.60	2.2 x10^{-5}	-7.5 x10^{-7}
6	0.07	0.09*	0.03	3423*	-368	-1.7 x10^{-4}	0.65	0.002	-1.2 x10^{-6}
7	0.02	0.04	0.02	2079	-313	-0.01	0.76	7.7 x10^{-4}	-2.6 x10^{-7}
8	0.05	0.05	0.05	2049	-394	-0.02	0.83	2.5 x10^{-4}	-7.6 x10^{-7}
9	0.07	0.07	0.04	2963*	-438	-0.01	0.55	0.001	-1.1 x10^{-6}

Adjusted for age, sex, type 2 diabetes and, where appropriate, total intracranial volume *$p<0.002$ with Bonferroni correction for multiple comparisons

Supplementary Table 6 Inner Plexiform Layer Thickness – associations with cognition, brain structure, global cerebral blood flow and glucose uptake

Thickness of Early Treatment of Diabetic Retinopathy Study segment (μm)	Memory β	Speed β	Attention β	Gray Matter Volume (ml) β	White Matter Hyperintensity Volume (ml) β	Presence of Cerebral Microbleed β	Total Cerebral Blood Flow (ml/min/100g) β	Mean Fractional Anisotropy β	Mean Mean Diffusivity (mmx10^{-7}) β
Right eye									
1	-0.08	-0.10	0.002	-1890	-100	0.002	0.82	8.3 x10^{-5}	-6.5 x10^{-7}
2	0.02	0.04	0.02	2039	-252	-3.8 x10^{-5}	1.05	-9.7 x10^{-5}	-6.5 x10^{-7}
3	0.007	0.04	0.02	2332	-176	-0.003	1.14	-4.7 x10^{-5}	-1.2 x10^{-6}
4	0.01	0.02	0.01	2130	-266	-8.7 x10^{-4}	0.91	1.6 x10^{-4}	-1.1 x10^{-6}
5	-0.02	1.9 x10^{-4}	0.006	1644	-116	-0.003	1.36*	-4.6 x10^{-4}	-8.4 x10^{-7}
6	0.04	0.05	0.04	2486	-574	3.0 x10^{-4}	0.37	0.002	-1.4 x10^{-6}
7	0.06	0.07	-0.002	2345	-517	-0.009	0.30	0.002	-1.1 x10^{-7}
8	0.09	0.07	-0.008	2412	-279	-0.003	0.90	7.9 x10^{-4}	-9.0 x10^{-8}
9	-0.02	0.04	0.10*	3407	-385	-1.5 x10^{-4}	1.49	2.7 x10^{-4}	-7.9 x10^{-7}
Left eye									
1	-0.09*	-0.01	-0.01	-1936	-115	-0.001	0.29	-6.7 x10^{-4}	-2.1 x10^{-8}
2	0.002	0.05	0.05	2095	-313	-0.004	0.88	6.1 x10^{-4}	-1.1 x10^{-6}
3	-0.03	0.01	0.01	2063	-253	-0.005	0.82	8.3 x10^{-4}	-1.5 x10^{-6}
4	-0.02	0.02	0.03	1849	-217	-0.003	1.00	2.5 x10^{-4}	-1.1 x10^{-6}
5	0.02	0.04	0.03	2335	-281	-0.007	0.95	5.0 x10^{-4}	-1.5 x10^{-6}
6	0.01	0.11	0.09	3904	-511	0.01	0.69	0.002	-9.4 x10^{-7}
7	-0.03	0.04	0.06	3171	-389	-0.009	0.96	1.5 x10^{-4}	-1.6 x10^{-6}
8	-0.05	0.02	0.08	865	-494	-0.02	0.59	2.5 x10^{-4}	-1.2 x10^{-6}
9	0.07	0.09	0.04	3996	-597	0.002	0.71	0.85	0.002

Adjusted for age, sex, type 2 diabetes and, where appropriate, total intracranial volume *p<0.002 with Bonferroni correction for multiple comparisons

Supplementary Table 7 Inner Nuclear Layer Thickness – associations with cognition, brain structure, global cerebral blood flow and glucose uptake

Thickness of Early Treatment of Diabetic Retinopathy Study segment (µm)	Memory β	Speed β	Attention β	Gray Matter Volume (ml) β	White Matter Hyperintensity Volume (ml) β	Presence of Cerebral Microbleed β	Total Cerebral Blood Flow (ml/min/100g) β	Mean Fractional Anisotropy β	Mean Mean Diffusivity (mmx10^{-7}) β
Right eye									
1	-0.06	-0.01	-0.01	-2254	-147	3.0 x10^{-4}	0.02	-1.1 x10^{-5}	-6.8 x10^{-8}
2	0.04	0.07	-0.005	2131	16	0.004	0.52	-4.3 x10^{-4}	-3.3 x10^{-7}
3	0.02	0.06	0.09*	1443	-196	2.5 x10^{-4}	1.00	2.1 x10^{-4}	-3.0 x10^{-7}
4	-0.007	0.03	-0.02	1176	-137	0.001	0.51	-3.0 x10^{-4}	-3.2 x10^{-7}
5	-0.05	0.05	0.03	2366	-132	0.003	0.42	-0.001	9.3 x10^{-8}
6	-0.002	0.09	0.01	3202	-118	0.02	0.18	-3.3 x10^{-4}	-8.7 x10^{-7}
7	0.008	0.10	0.01	3119	-385	0.02	0.41	4.9 x10^{-4}	-1.2 x10^{-6}
8	0.03	0.09	-0.002	1405	-137	0.006	1.37	-7.6 x10^{-4}	-4.6 x10^{-7}
9	-0.004	0.04	0.05	1069	-47	0.002	0.39	-0.002	-1.1 x10^{-6}
Left eye									
1	-0.07*	-0.01	-0.01	-1712	-119	-0.002	-0.13	-0.004	1.1 x10^{-7}
2	0.02	0.08*	-0.003	2588*	-99	0.005	0.54	4.0 x10^{-4}	-8.9 x10^{-7}
3	-0.05	0.03	0.07*	508	-72	-0.01	0.93*	-4.5 x10^{-4}	-2.4 x10^{-7}
4	-0.03	0.04	0.04	1611	-74	-0.008	0.85	-5.2 x10^{-5}	-7.6 x10^{-7}
5	-0.08*	0.02	0.04*	1107	-182	0.006	0.45	1.7 x10^{-4}	-7.2 x10^{-7}
6	0.006	0.08	0.002	956	331	5.8 x10^{-4}	0.26	-0.002	-8.0 x10^{-8}
7	3.2 x10^{-4}	0.08	0.03	1269	-197	-0.002	0.94	-0.001	-1.1 x10^{-7}
8	-0.09	-0.02	0.04	-970	-182	-0.009	1.07	-0.002	5.7 x10^{-8}
9	-0.04	0.07	0.03	930	-278	0.01	0.31	3.2 x10^{-4}	-8.7 x10^{-7}

Adjusted for age, sex, type 2 diabetes and, where appropriate, total intracranial volume *$p<0.002$ with Bonferroni correction for multiple comparisons

Supplementary Table 8 Outer Plexiform Layer Thickness – associations with cognition, brain structure, global cerebral blood flow and glucose uptake

Thickness of Early Treatment of Diabetic Retinopathy Study segment (μm)	Memory β	Speed β	Attention β	Gray Matter Volume (ml) β	White Matter Hyperintensity Volume (ml) β	Presence of Cerebral Microbleed β	Total Cerebral Blood Flow (ml/min/100g) β	Mean Fractional Anisotropy β	Mean Mean Diffusivity (mmx10^{-7}) β
Right eye									
1	-0.03	-0.005	-0.05*	-1163	48.0	-0.001	-0.20	-1.6 x10^{-4}	3.1 x10^{-7}
2	-0.01	0.01	-0.01	525	33.9	-0.002	-0.16	3.6 x10^{-4}	-3.0 x10^{-7}
3	-0.05	-0.01	-0.01	-125	-104.7	-0.005	0.17	1.8 x10^{-4}	-1.4 x10^{-7}
4	-0.008	-0.02*	0.008	-184	-72	-0.004	0.17	-1.8 x10^{-4}	3.4 x10^{-7}
5	0.005	-0.02	0.009	536	26	-0.003	0.21	2.5 x10^{-4}	-1.8 x10^{-7}
6	-1.1 x10^{-4}	-2.8 x10^{-4}	-0.004	-135	-3.7	-3.4 x10^{-4}	-0.03	8.3 x10^{-5}	3.9 x10^{-8}
7	-0.07	-0.02	-0.007	-1111	-104	0.003	-0.30	0.001	-3.1 x10^{-8}
8	0.01	-0.02	0.03	-200	-295	-0.01	0.86	6.4 x10^{-4}	1.0 x10^{-6}
9	0.007	-0.03	0.02	-184	-68	-0.02	0.70	1.6 x10^{-4}	2.8 x10^{-7}
Left eye									
1	-0.006	0.03	-0.05	1585	97	0.01	-0.19	5.7 x10^{-4}	-1.0 x10^{-6}
2	0.01	0.02	-0.02	844	70	0.004	-0.08	3.0 x10^{-4}	-4.4 x10^{-4}
3	0.03	0.02	-0.03	2346	55	0.005	0.57	0.001	-8.7 x10^{-7}
4	-0.02	-0.02	0.003	-292	-49	-0.001	0.28	8.1 x10^{-6}	1.8 x10^{-7}
5	-0.02	-0.006	-0.02	344	14	0.008	-0.23	4.7 x10^{-4}	-8.4 x10^{-7}
6	0.04	0.06	-0.06	996	102	0.02	0.09	9.9 x10^{-4}	-1.1 x10^{-6}
7	0.03	0.02	-0.02	1868	-185	0.01	0.44	0.002	1.6 x10^{-6}
8	-0.07	-0.11*	0.03	-1067	-94	0.01	0.69	-4.1 x10^{-4}	7.8 x10^{-7}
9	-0.04	-0.03	-0.02	54	58	0.02	0.43	3.4 x10^{-4}	-5.4 x10^{-7}

Adjusted for age, sex, type 2 diabetes and, where appropriate, total intracranial volume *$p<0.002$ with Bonferroni correction for multiple comparisons

Supplementary Table 9 Outer Nuclear Layer Thickness – associations with cognition, brain structure, global cerebral blood flow and glucose uptake

Thickness of Early Treatment of Diabetic Retinopathy Study segment (μm)	Memory β	Speed β	Attention β	Gray Matter Volume (ml) β	White Matter Hyperintensity Volume (ml) β	Presence of Cerebral Microbleed β	Total Cerebral Blood Flow (ml/min/100g) β	Mean Fractional Anisotropy β	Mean Mean Diffusivity (mmx10^{-7}) β
Right eye									
1	0.008	0.008	0.008	355	9.6	-0.01*	0.25	-8.2 x10^{-5}	1.5 x10^{-7}
2	0.008	-0.006	0.01	-360	-27.2	-0.005	0.24	-3.0 x10^{-4}	3.2 x10^{-7}
3	0.02	0.009	0.01	-12.2	41.0	-0.007	0.12	-1.7 x10^{-4}	3.2 x10^{-7}
4	0.008	0.02	-0.002	244	61.6	-0.002	-0.02	9.7 x10^{-5}	-2.1 x10^{-7}
5	-0.008	0.02	0.009	-165	-8.2	-0.009	0.17	-2.1 x10^{-4}	2.9 x10^{-7}
6	0.009	0.01	0.02	223	-60.9	-0.003	0.15	-4.0 x10^{-5}	2.8 x10^{-7}
7	2.5 x10^{-4}	0.02	0.02	-235	-50.7	-0.004	-0.11	-1.2 x10^{-4}	3.3 x10^{-7}
8	-0.02	-0.01	9.3 x10^{-4}	-237	13.0	7.7 x10^{-4}	-0.15	-2.0 x10^{-4}	-1.0 x10^{-7}
9	-0.006	0.02	0.02	253	2.5	-0.004	-0.04	-1.1 x10^{-4}	2.0 x10^{-7}
Left eye									
1	0.02	0.006	0.009	-85	-0.29	-0.01*	0.22	6.8 x10^{-5}	2.0 x10^{-7}
2	-0.01	-0.01	0.03*	-569	-60.3	-0.01	0.16	-9.0 x10^{-5}	4.2 x10^{-7}
3	0.002	0.005	0.01	-236	23.6	-0.008	0.10	-4.0 x10^{-4}	5.1 x10^{-7}
4	-0.002	0.02	0.01	166	13.0	-0.004	-0.009	-8.0 x10^{-5}	8.5 x10^{-8}
5	0.02	0.01	0.02	118	-12.0	-0.01	0.21	7.2 x10^{-5}	3.4 x10^{-7}
6	-0.02	0.004	0.05*	-150	-83.0	-0.007	-0.06	-3.7 x10^{-5}	5.4 x10^{-7}
7	-0.008	0.01	0.03	-240	-33.2	-0.004	-0.11	-2.0 x10^{-4}	5.1 x10^{-7}
8	-0.005	0.03	0.01	115	-63.8	-0.003	-0.16	2.2 x10^{-4}	-9.1 x10^{-8}
9	0.01	0.03	0.02	-54	-80.0	-0.005	-0.09	2.0 x10^{-4}	1.3 x10^{-7}

Adjusted for age, sex, type 2 diabetes and, where appropriate, total intracranial volume *p<0.002 with Bonferroni correction for multiple comparisons

Supplementary Table 10 Retinal Pigment Epithelium Layer Thickness – associations with cognition, brain structure, global cerebral blood flow and glucose uptake

Thickness of Early Treatment of Diabetic Retinopathy Study segment (μm)	Memory β	Speed β	Attention β	Gray Matter Volume (ml) β	White Matter Hyperintensity Volume (ml) β	Presence of Cerebral Microbleed β	Total Cerebral Blood Flow (ml/min/100g) β	Mean Fractional Anisotropy β	Mean Mean Diffusivity (mmx10^{-7}) β
Right eye									
1	0.02	0.06	-0.04	-1152	402	0.004	-0.60	-0.001	-2.2 x10^{-7}
2	0.10	0.22*	-0.12	3723	503	-0.02	1.55	-8.5 x10^{-4}	9.1 x10^{-7}
3	0.04	0.13	-0.12	2266	400	0.02	-0.12	-4.9 x10^{-4}	-7.2 x10^{-7}
4	0.05	0.09	-0.13*	40	631	-0.01	1.10	-0.001	8.3 x10^{-7}
5	0.06	0.15	-0.17*	1275	45.6	0.005	0.29	5.2 x10^{-4}	-3.5 x10^{-7}
6	0.09	0.19*	-0.12	6916	437	0.09	-0.96	-3.1 x10^{-4}	-9.3 x10^{-7}
7	-0.11	-0.07	-0.16	720	352	0.08	-0.92	-0.001	1.2 x10^{-7}
8	-0.10	-0.05	-0.07	889	278	0.03	-0.41	-0.001	-7.5 x10^{-7}
9	0.08	0.10	-0.20	2205	594	-0.03	1.35	-0.002	2.4 x10^{-6}
Left eye									
1	0.05	0.09	-0.10	3892	56	0.02	-0.82	0.003	-7.4 x10^{-7}
2	-0.02	0.004	0.007	-280	-55	-0.002	-0.01	5.4 x10^{-5}	-1.9 x10^{-8}
3	-0.10	0.10	0.002	580	-230	0.01	-0.14	0.002	-8.4 x10^{-8}
4	0.10	0.21	-0.14	3640	756	0.06	-0.95	2.3 x10^{-4}	-3.5 x10^{-7}
5	0.01	0.16*	-0.10	2578	460	0.04	-0.78	0.002	-5.6 x10^{-7}
6	-0.10	0.08	-0.04	-116	165	0.06	-0.45	-2.7 x10^{-4}	1.2 x10^{-6}
7	0.09	0.15	-0.17	3250	416	0.04	0.79	0.002	1.4 x10^{-6}
8	-0.04	0.01	0.01	-375	-97	-0.002	-0.08	1.3 x10^{-4}	-9.0 x10^{-8}
9	-0.002	0.13	-0.17	-213	606	0.10	-0.77	-5.6 x10^{-5}	1.1 x10^{-6}

Adjusted for age, sex, type 2 diabetes and, where appropriate, total intracranial volume *$p<0.002$ with Bonferroni correction for multiple comparisons

Supplementary Table 11 Total retinal thickness – associations with cognition, brain structure, global cerebral blood flow and glucose uptake

Thickness of Early Treatment of Diabetic Retinopathy Study segment (μm)	Memory β	Speed β	Attention β	Gray Matter Volume (ml) β	White Matter Hyperintensity Volume (ml) β	Presence of Cerebral Microbleed β	Total Cerebral Blood Flow (ml/min/100g) β	Mean Fractional Anisotropy β	Mean Diffusivity (mmx10^{-7}) β
Right eye									
1	-0.009	0.004	-0.005	-203	3.5	-0.004	0.12	-5.7 x10^{-5}	2.2 x10^{-8}
2	-0.003	0.006	0.007	238	-29	-0.002	0.10	-1.1 x10^{-4}	4.1 x10^{-8}
3	-5.9 x10^{-4}	0.01	0.006	411	-18.3	-0.005	0.26	-5.3 x10^{-5}	-4.4 x10^{-8}
4	0.003	0.01	0.003	478	-17.3	-0.003	0.27*	-8.6 x10^{-5}	-4.7 x10^{-8}
5	-0.003	0.009	0.005	511	-26.3	-0.005	0.31*	-8.7 x10^{-5}	-6.9 x10^{-8}
6	0.007	0.02	0.012	908*	-89.8	-5.0 x10^{-4}	0.18	2.9 x10^{-4}	-1.9 x10^{-7}
7	0.003	0.02	0.008	764	-63.7	-5.2 x10^{-4}	0.17	-2.7 x10^{-5}	-7.9 x10^{-8}
8	-0.01	-0.005	0.002	310	-6.1	4.7 x10^{-4}	0.11	-1.6 x10^{-4}	-1.4 x10^{-7}
9	0.001	0.012	0.017	645	-47.8	-0.003	0.23	1.2 x10^{-5}	-7.5 x10^{-8}
Left eye									
1	-0.006	0.001	0.002	-102	-14.5	-0.001	0.01	6.7 x10^{-6}	-1.0 x10^{-8}
2	-0.004	0.001	0.002	-16	-14.5	-6.1 x10^{-4}	0.01	2.0 x10^{-5}	-1.7 x10^{-8}
3	-0.005	0.001	0.002	-18	-16.5	-9.7 x10^{-4}	0.02	2.5 x10^{-5}	-2.2 x10^{-8}
4	-0.005	0.010	0.009	422	-31.0	-0.003	0.21	9.1 x10^{-5}	-1.1 x10^{-7}
5	-0.007	0.003	0.004	28	-28.5	-0.001	0.06	5.8 x10^{-5}	-4.9 x10^{-8}
6	2.7 x10^{-5}	0.02	0.011	890*	-57.2	-5.4 x10^{-5}	0.22	3.8 x10^{-4}	6.5 x10^{-9}
7	-0.005	0.01	0.009	735	-49.8	-0.002	0.22	5.5 x10^{-5}	7.6 x10^{-8}
8	-0.006	0.002	0.003	-35	-25.9	-0.001	0.01	4.3 x10^{-5}	-2.8 x10^{-8}
9	0.005	0.016	0.010	650	-58.1	-3.7 x10^{-4}	0.16	2.6 x10^{-4}	-1.2 x10^{-7}

Adjusted for age, sex, type 2 diabetes and, where appropriate, total intracranial volume *$p<0.002$ with Bonferroni correction for multiple comparisons
